# Repositioning Aspirin to Treat Lung and Breast Cancers and Overcome Acquired Resistance to Targeted Therapy

**DOI:** 10.3389/fonc.2019.01503

**Published:** 2020-01-14

**Authors:** Ling Li, Mengdi Hu, Tao Wang, Hongzhuan Chen, Lu Xu

**Affiliations:** ^1^Department of Pharmacology and Chemical Biology, Shanghai Jiao Tong University School of Medicine, Shanghai, China; ^2^Institute of Interdisciplinary Integrative Biomedical Research, Shanghai University of Traditional Chinese Medicine, Shanghai, China

**Keywords:** aspirin, lung cancer, breast cancer, targeted therapy, EGFR TKIs, tamoxifen, CMap, reposition

## Abstract

**Background:** The major limitation of targeted cancer therapy is development of acquired resistance. Intratumoral heterogeneity and coexist of multiple resistance mechanisms make combination therapies targeting one specific mechanism inefficient.

**Methods:** Transcriptional signature obtained from GEO was used to reposition FDA-approved drugs to treat lung and breast cancers as well as overcome acquired resistance to EGFR TKIs in lung cancer and to tamoxifen in breast cancer via CMap. *In vitro* and *in vivo* models were used to examine candidate drugs for their anti-cancer and anti-resistance efficacy and underlying mechanisms.

**Results:** We found that aspirin, the most commonly used drug, not only inhibited proliferation and promoted apoptosis of cancer cells, but also delayed and overcame acquired resistance to targeted therapy using *in vitro* and *in vivo* models. The underlying mechanism could be attributed to enhanced cancer stemness and activated NF-κB signaling in acquired resistant tumors, both of which were suppressed by aspirin and rendered resistant tumors more sensitive to aspirin.

**Conclusions:** Our data identify aspirin as a potential candidate for combination therapy for lung and breast cancers.

## Background

Cancer is the leading cause of death worldwide. Treatment for cancer includes surgery, chemotherapy, and radiation therapy. Recently, the emergence of targeted therapy, which directly targets molecules that are uniquely or abnormally expressed in cancer cells, has changed dramatically the treatment of cancer. For example, tamoxifen was the first targeted therapy using estrogen receptor (ER) as the target, which is present in about 80% of all breast cancer (ER-positive), and reduces greatly the incidence of breast cancer death and recurrence in ER-positive patients. Epidermal growth factor receptor (EGFR) is another effective target for treatment of many epithelial cancers, especially non-small cell lung cancer in which 10–55% patients have EGFR mutation. EGFR tyrosine kinase inhibitors (TKIs) have been approved for the first line-treatment for EGFR-mutant lung cancer patients. However, the limitation of targeted therapy is acquired resistance developed during the treatment course. The one major mechanism of acquired resistance to targeted therapy is the alterations in the target itself (on-target), such as second mutation T790M in EGFR in EGFR-mutant lung cancer or loss of ER function/expression in ER+ breast cancer. Next-generation drugs targeting altered targets need to be developed to overcome on-target acquired resistance. The other type of mechanisms is compensatory activation of downstream or parallel signaling pathways (off-target), such as RAS-RAF-MEK-ERK, PI3K-PTEN-AKT-mTOR, IGF1R pathway, NF-κB pathway et al., which were common in both lung and breast cancer with acquired resistance to respective targeted therapy (1–4) and could be overcome by combination therapies. Unfortunately, intratumoral heterogeneity which has been linked to treatment resistance and tumor recurrence as well as coexist of multiple resistance mechanisms render combination therapy targeting one specific molecule or pathway inefficient (5, 6).

Drug repositioning or repurposing is to apply an existing drug for another indication than it was originally approved for and has recently gained popularity as an alternative strategy to *de novo* drug synthesis, which is a time-consuming and costly process (7, 8). Systematic repurposing approaches can be subdivided into computational approaches and experimental approaches, both of which are often used synergistically. Signature matching is one of the most commonly used computational approaches, which is based on the comparison of the signature of a drug against that of another drug or disease. Connectivity map (CMap, http://www.broad.mit.edu/cmap/) is a transcriptional expression database containing compound-perturbed gene expression profiles of cultured human cell lines. Of the 1309 compounds included in CMap, most of them are currently used in clinic or well-developed. By comparison of the transcriptome of human cells treated with compound with that of a disease, which can be easily accessed through public databases like Gene Expression Omnibus (GEO), some old drugs have been successfully repositioned. For example, using glioblastoma gene signatures collected from GEO to query CMap and then cell-based screening of 65 candidate drugs, Cheng et al. found that thioridazine, a DRD2 antagonist/antipsychotic drug, had anticancer stem cell effects (9). A phase I trial has been conducted on acute myeloid leukemia (AML) patients to evaluate thioridazine in combination with cytarabine and preliminary results suggest that DRD2 represents a potential therapeutic target for AML (10).

Aspirin is the most common used non-steroidal anti-inflammatory drug (NSAID) and daily intake of 75–1,200 mg aspirin per day has been reported to reduce the incidence of colorectal cancer (11). However, the anticancer mechanisms of aspirin, the most commonly used drug and emerging candidate of drug repositioning, have not been yet clear.

The aim of this study was to reposition FDA-approved drugs as part of combination therapy to overcome acquired resistance to EGFR TKIs in lung cancer and to tamoxifen in breast cancer, targeting their common mechanisms underlying off-target acquired resistance. We searched GEO database to obtain gene signatures associated with lung/breast cancer and acquired resistance to EGFR TKIs/tamoxifen to query CMap. The top-ranked candidate aspirin was examined for its anticancer and antiresistance effects on *in vitro* cells and *in vivo* animal models and the underlying mechanisms were also explored.

## Materials and Methods

### Reagents

Gefitinib, osimertinib and tamoxifen were purchased from Selleck (Shanghai, China). Aspirin was purchased from Sangon Biotech (Shanghai, China). Gefitinib, osimertinib, tamoxifen, and aspirin were dissolved in DMSO.

### Drug Screening via the CMap

Nine datasets from GEO (GSE19804, GSE42568, GSE15852, GSE10797, GSE7670, GSE74575, GSE38310, GSE67916, and GSE122005) were used in this study, all of which were generated using Affymetrix HG-U133A gene chips. Two-fold change with *P* < 0.05 was used as the cut-off criterion for up and down probe sets, which were used to query CMap. Compounds with *P* < 0.05 and enrichment score < -0.5 were retained.

### Cell Culture and Establishment of Resistant Cancer Cell Lines *in vitro*

HCC827, 16HBE, and MCF-7 cells were purchased from Cell Bank of Type Culture Collection of the Chinese Academy of Sciences (Shanghai, China). MCF-10A cells were gifts from Dr. Zhaoyuan Hou in Shanghai Jiao Tong University School of Medicine. The cell lines were cultured under standard condition and tested by certified third-party laboratories for authenticity using short tandem repeat analysis and examined for mycoplasma regularly. Gefitinib-, osimertinib-, and tamoxifen-resistant cells were established by the stepwise escalation method and maintained as previously described (12). Briefly, parental cells were cultured with stepwise escalation of concentration of gefitinib, osimertinib or tamoxifen from 5 to 5 μM over 6 months. Resistant cell lines are capable of proliferating normally in the presence of 5 μM gefitinib, osimertinib or tamoxifen. Cell viability was used to confirm resistance after allowing the cells to grow in drug-free medium for 5–7 days. Upon confirming resistance, resistant cell lines were cultured without gefitinib, osimertinib, or tamoxifen and their resistance was examined periodically.

### Cell Viability Assay

Cell viability was determined using the Cell Counting Kit-8 (CCK-8) colorimetric assay (Dojindo, Shanghai, China) and the IncuCyte ZOOM® system (Essen BioScience) as previously described (12). Briefly, for CCK8 assay, cells were seeded at a density of 2,000–3,000 cells/well in 96-well plates. After incubated with serum-free DMEM for 24 h, the cells were treated with indicated concentrations of drug for 48–72 h. Cells treated with solvent (DMSO) were used as a control, with viability set at 100%. For IncuCyte assay, 3,000 cells were seeded into 96-well plates containing DMEM supplemented with 10% FBS in the presence or absence of indicated concentrations of drug. The plates were placed into an IncuCyte Zoom (Essen Bioscience) that automatically takes phase-contrast images of each well every 2 h over the course of 2–5 days and utilizes software to measure confluence as a proxy for cell viability.

### Western Blot and Immunofluorescence Analyses

The expression levels of proteins were examined by Western blot and immunofluorescence staining analyses as previously described (12). The list of antibodies used is available in Table S2.

### Colony Formation Assay

A total of 800–1,000 viable cells were placed in six-well plates and cultured in complete medium for 2–3 weeks. Colonies were fixed, stained with crystal violet and then counted.

### Mouse Xenograft Models, Combination Treatment, and Tumorigenic Assay

Athymic BALB/c nude mice were purchased from Shanghai Laboratory Animal Center (Chinese Academy of Sciences, Shanghai, China) and housed in environmentally controlled, specific pathogen-free conditions for 1 week before the study. All experimental procedures were reviewed and approved in accordance with the guidelines for the care and use of laboratory animals at Shanghai Jiao Tong University.

To establish mouse xenograft models, same amount of indicated tumor cells was injected subcutaneously into both flanks of each mouse. The tumor volume was measured after 1 week from injection and then every other day or twice a week. Tumor volumes (mm^3^) were calculated as length × width^2^/2.

For combination treatment experiments, when the volumes of xenograft tumors reached ~200 mm^3^, mice were given daily PBS, 12.5 mg/kg gefitinib, 100 mg/kg aspirin, or combination of 12.5 mg/kg gefitinib and 100 mg/kg aspirin by garage. At the end of experiments, mice were sacrificed and tumors were dissected, weighted and photographed.

For tumorigenic assay, HCC827 cells were treated with aspirin for 12 h and then different amount of viable cells (1 × 10^6^, 5 × 10^5^, 2 × 10^5^, 1 × 10^5^) in 50 μl PBS were injected subcutaneously into each mouse. Xenograft tumor initiation and growth were examined every 5 days.

### Statistical Analysis

All data are presented as the mean±SEM. Statistical analysis was conducted using GraphPad Prism 5.0 software (La Jolla, CA, USA). Differences between groups were examined using Student's *t*-test. Differences were considered significant if *P* was <0.05.

## Results

### Using Gene Signatures to Identify Drugs for Lung and Breast Cancers via CMap

**Figure 1:**
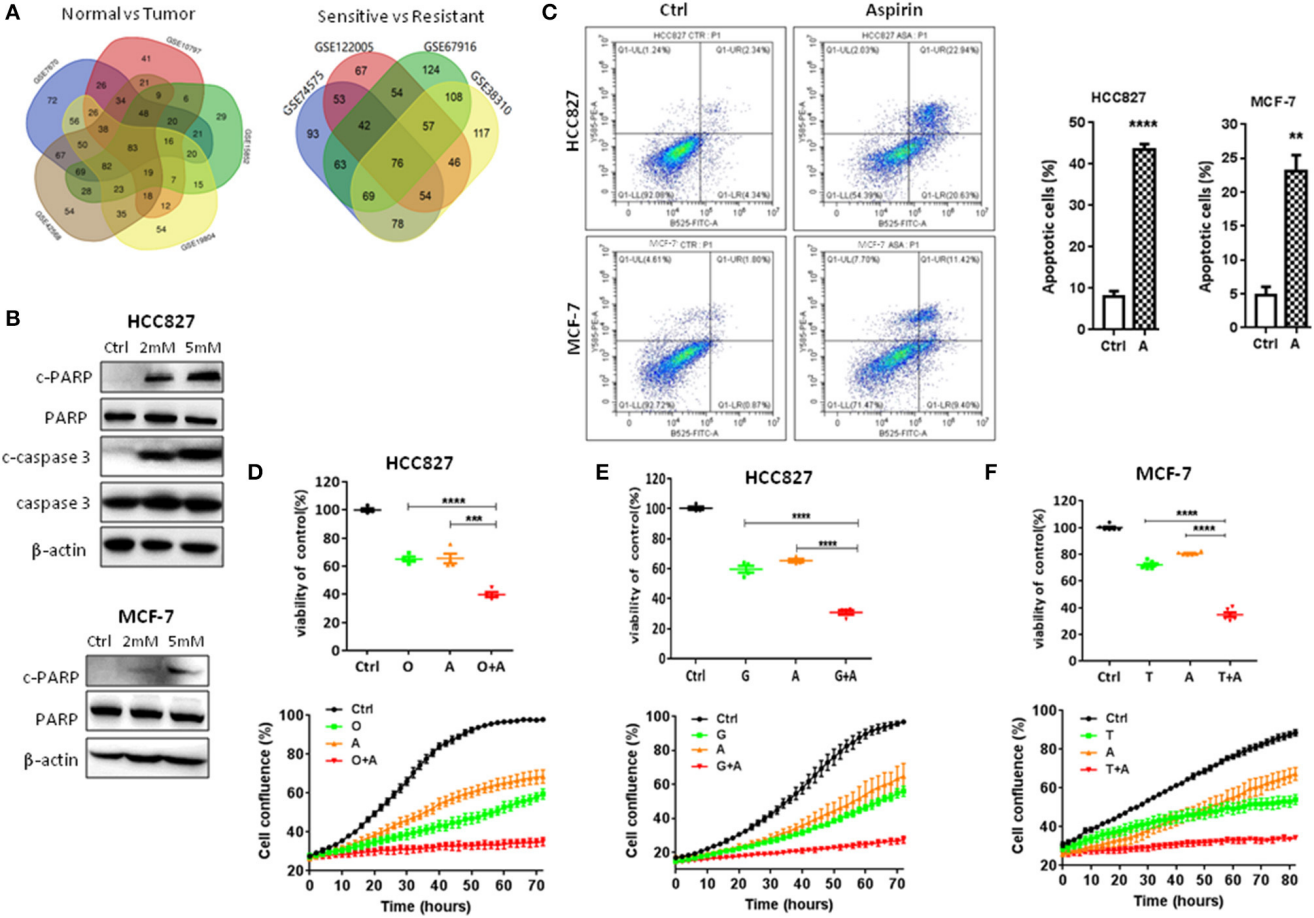
Aspirin inhibited proliferation and promoted apoptosis of lung and breast cancer cells. **(A)** The Venn diagram represents the number of drugs using five data sets (GSE19804, GSE42568, GSE15852, GSE10797, and GSE7670) or four data sets (GSE74575, GSE38310, GSE67916, GSE122005) to query the CMap. **(B)** Western blot analysis of cleaved-caspase 3 (c-caspase 3), caspase 3, cleaved-PARP (c-PARP), and PARP in HCC827 and MCF-7 cells treated with indicated concentrations of aspirin for 72 h. β-actin was used as loading control. **(C)** Flow cytometry analysis of apoptosis of HCC827 treated with 2 mM aspirin and of MCF-7 cells treated with 2 mM aspirin for 72 h (left panels). Quantitative analysis of apoptotic cells identified by flow cytometry (right panels). Data were obtained from three independent experiments. **(D–F)** Cell viability by CCK8 assay (upper panels) and IncuCyte growth curves (lower panels) of indicated cells treated with 10 nM osimertinib (O), 10 nM gefitinib (G), 2 mM aspirin (A), 2 μM tamoxifen (T), O+A, G+A, or T+A for 72 h as indicated. The data are presented as the means ± SEM. Student's *t*-test was used. *P < 0.05; **P < 0.01; ***P < 0.001; ****P < 0.0001.

It is currently acknowledged that transcriptional programs can be used to identify therapeutic targets to treat cancer. If a drug treatment could reverse the gene signature of a certain disease, it might have the potential to treat the disease. The Connectivity Map (CMap) database comprised a large reference collection of gene expression profiles from cultured human cells treated with 1,309 drugs. The database can be queried with a gene signature of interest to identify those drugs that induce desired gene expression changes. In order to identify drugs to treat the two most common cancers lung and breast cancers, as well as overcome acquired resistance to targeted therapy, first, we searched the Gene Expression Omnibus (GEO) database for lung cancer or breast cancer vs. respective normal tissue, EGFR TKI-sensitive vs. acquired resistant lung cancer, and tamoxifen-sensitive vs. acquired resistant breast cancer. We obtained five datasets for cancer vs. normal and four for targeted-therapy sensitive vs. acquired resistant. The data sources were summarized in Tables 1, 2 and data analysis was described in the Material and methods section. All nine datasets were published previously (13–21). Then, differentially expressed genes from each dataset were individually queried with CMap. As shown in Figure 1A, intersection of five normal vs. cancer datasets and four targeted-therapy sensitive vs. acquired resistant datasets yielded 83 and 76 drugs, respectively. Both of those two groups of drugs contained the same 12 FDA-approved drugs and the names of these drugs were listed in Table S1.

**Table 1:** Summary of five datasets (normal vs. tumor samples) used for CMap analysis.

**GEO #**	**GSE7670**	**GSE10797**	**GSE15852**	**GSE19804**	**GSE42568**
Pubmed ID	17540040	18373191	20097481	20802022	23740839
Up probe sets #	21	7	18	9	4
Down probe sets #	109	110	47	47	103
Normal #	27	10	43	60	17
Tumor #	27	56	43	60	104

**Table 2:** Summary of four datasets (sensitive vs. resistant cells) used for CMap analysis.

**GEO #**	**GSE38310**	**GSE74575**	**GSE122005**	**GSE67916**
Pubmed ID	22751098	27108960	30609749	24882577
Up probe sets #	13	48	33	13
Down probe sets #	30	29	41	11
Sensitive #	3	3	3	8
Resistant #	3	3	3	10

### Aspirin Inhibited Proliferation and Promoted Apoptosis of Cancer Cells

First, we tested the effects of these drugs on the proliferation and apoptosis of lung and breast cancer cells. As shown in Figure 1B, in a dose-dependent manner, aspirin promoted apoptosis of lung cancer HCC827 and breast cancer MCF-7 cells by increasing the expression of cleaved PARP or caspase-3. Flow cytometry also showed significantly increased percentage of apoptotic cells when treated with aspirin (Figure 1C). Then, we treated HCC827 cells with EGFR TKIs (gefitinib or osimertinib) and MCF-7 cells with tamoxifen in combination with aspirin (A). Cell viability assays (Figures 1D–F, upper panels) and IncuCyte growth curves (Figures 1D–F, lower panels) both showed that combination of aspirin with targeted drugs dramatically inhibited proliferation of cancer cells. However, single agents including targeted therapies and aspirin, or combination of targeted therapies and aspirin had no effects on the proliferation of normal lung (16HBE) or breast epithelial cells (MCF-10A) (Figure S1).

### Aspirin Overcame Acquired Resistance to Targeted Therapy

**Figure 2:**
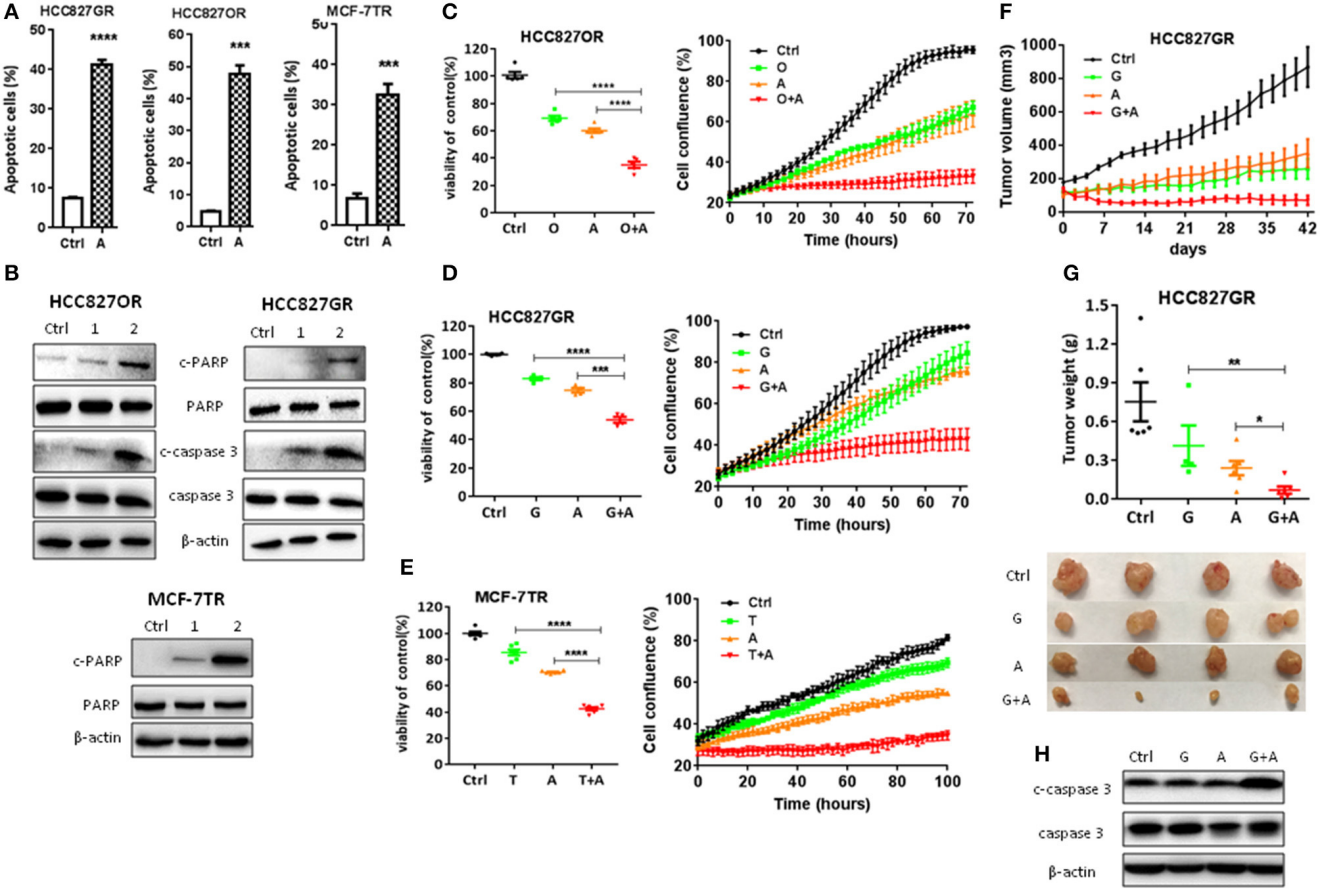
Aspirin overcame acquired resistance to targeted therapy in lung and breast cancers. **(A)** Quantitative analysis of apoptotic cells in the indicated cells treated with 1 mM aspirin for 72 h identified by flow cytometry. Data were obtained from three independent experiments. **(B)** Western blot analysis of c-caspase 3, caspase 3, c-PARP, and PARP in HCC827OR, HCC827GR, and MCF-7TR cells treated with indicated concentrations (mM) of aspirin for 72 h. **(C–E)** Cell viability by CCK8 assay (left panels) and IncuCyte growth curves (right panels) of indicated cells treated with 10 μM osimertinib (O), 10 μM gefitinib (G), 1 mM aspirin (A), 8 μM tamoxifen (T), O+A, G+A, or T+A for 72 h as indicated. **(F–H)** HCC827GR-derived xenograft tumors were treated with PBS, 12.5 mg/kg/day gefitinib (G), 100 mg/kg/day aspirin (A), or G+A. The growth curve **(F)**, and the weight and photographs of tumors **(G)**. **(H)** Western blot analysis of c-caspase 3 and caspase 3. β-actin was used as loading control. The data are presented as the means ± SEM. Student's *t*-test was used. *P < 0.05; **P < 0.01; ***P < 0.001; ****P < 0.0001.

Next, we determined to test the effects of aspirin on the acquired resistance to targeted therapy in lung and breast cancers. First, we established *in vitro* cell models of acquired resistance to targeted therapy by culturing sensitive cancer cells in targeted drugs with escalating concentration as we previously reported (12). Then we examined the effects of aspirin on the cell viability in normal epithelium cells, sensitive and resistant cells using CCK8 assay. As shown in Figures S2A,B, resistant cells such as HCC827GR, HCC827OR, and MCF-7TR cells were more sensitive to aspirin than their respective sensitive cells, HCC827 cells and MCF-7 cells, while sensitive cells were more sensitive to aspirin than normal epithelium cells, 16HBE and MCF-10A. Therefore, we chose 1 or 2 mM aspirin to treat resistant cells and 2 or 5 mM to treat sensitive cells in this study. We found that aspirin promoted apoptosis by increasing the percentages of apoptotic cells (Figure 2A and Figure S2C) and the expression of cleaved PARP and caspase-3 (Figure 2B). Similarly, we treated resistant cells with targeted drugs in combination with aspirin. Cell viability assays (Figures 2C–E, left panels) and IncuCyte growth curves (Figures 2C–E, right panels) both showed that combination of aspirin with targeted drugs dramatically inhibited proliferation of resistant cells. To extend our findings to *in vivo*, we subcutaneously injected HCC827GR cells into both flanks of nude mice. And when tumor cells formed solid, palpable tumor with an average volume of 150–200 mm^3^, we treated mice with gefitinib (12.5 mg/kg/day) in combination with aspirin (100 mg/kg/day). As shown in Figures 2F–H, combination therapy dramatically inhibited tumor growth (Figures 2F,G) and promoted apoptosis compared to single agent therapy (Figure 2H).

### Aspirin Delayed the Emergence of Acquired Resistance to Targeted Therapy

**Figure 3:**
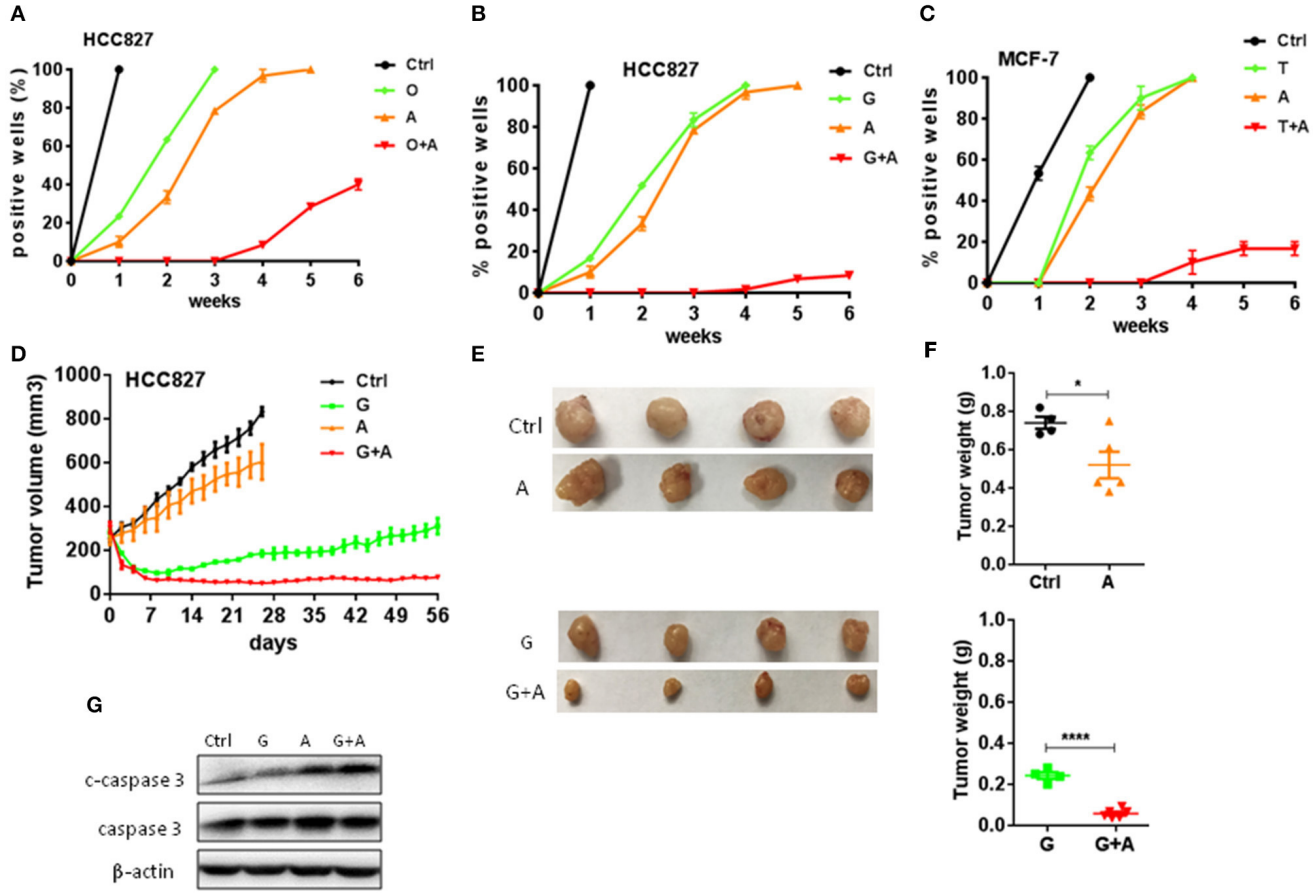
Aspirin delayed the emergence of acquired resistance to targeted therapy in lung and breast cancers. **(A–C)** The indicated cells (200–500/well) were seeded and treated in a 96-well plate, and wells of 50% or greater confluence were scored as positive weekly and graphed means ± SEM. Each experiment was repeated two times. **(A)** HCC827 cells were treated with 10 nM O, 2 mM A, or O+A. **(B)** HCC827 cells were treated with 10 nM G, 2 mM A, or G+A. **(C)** MCF-7 cells were treated with 2 μM T, 2 mM A, or T+A. **(D–F)** HCC827-derived xenograft tumors were treated with PBS, 12.5 mg/kg/day G, 100 mg/kg/day A, or G+A. The growth curve **(D)**, the photographs **(E)** and the weight of tumors **(F)**. **(G)** Western blot analysis of c-caspase 3 and caspase 3. β-actin was used as loading control. The data are presented as the means ± SEM. Student's *t*-test was used. *P < 0.05; ****P < 0.0001.

To determine whether aspirin could prevent or delay the occurrence of acquired resistance to targeted therapy, we assessed the emergence of acquired resistance to targeted drugs. Low confluence cells (200–500/well) were seeded and treated in a 96-well plate, and wells of 50% or greater confluence were scored as positive weekly (22). We found that gefitinib- and osimertinib-resistant colonies began to appear within 1 week, while tamoxifen-resistant colonies appeared within 2 weeks (Figures 3A–C). Aspirin alone didn't delay the emergence of resistance while combination of aspirin with targeted drugs significantly delayed the emergence of acquired resistance and reduced the incidence of resistant colonies (Figures 3A–C). To recapitulate the *in vitro* results *in vivo*, nude mice harboring HCC827-xenograft tumors were treated with gefitinib (12.5 mg/kg/day), aspirin (100 mg/kg/day), or combination of thereof. During the course of treatment, aspirin alone slightly inhibited tumor growth while gefitinib alone or in combination with aspirin led to significant tumor regression (Figures 3D–F). However, gefitinib alone was not able to prevent tumor regrowth after about 3-week treatment, indicating emergence of acquired resistance, while combination of gefitinib with aspirin effectively suppressed tumor regrowth up to 8 weeks. Moreover, combination therapy dramatically promoted apoptosis compared to single agent therapy (Figure 3G).

### Aspirin Suppressed Cancer Stemness of Lung and Breast Cancers

**Figure 4:**
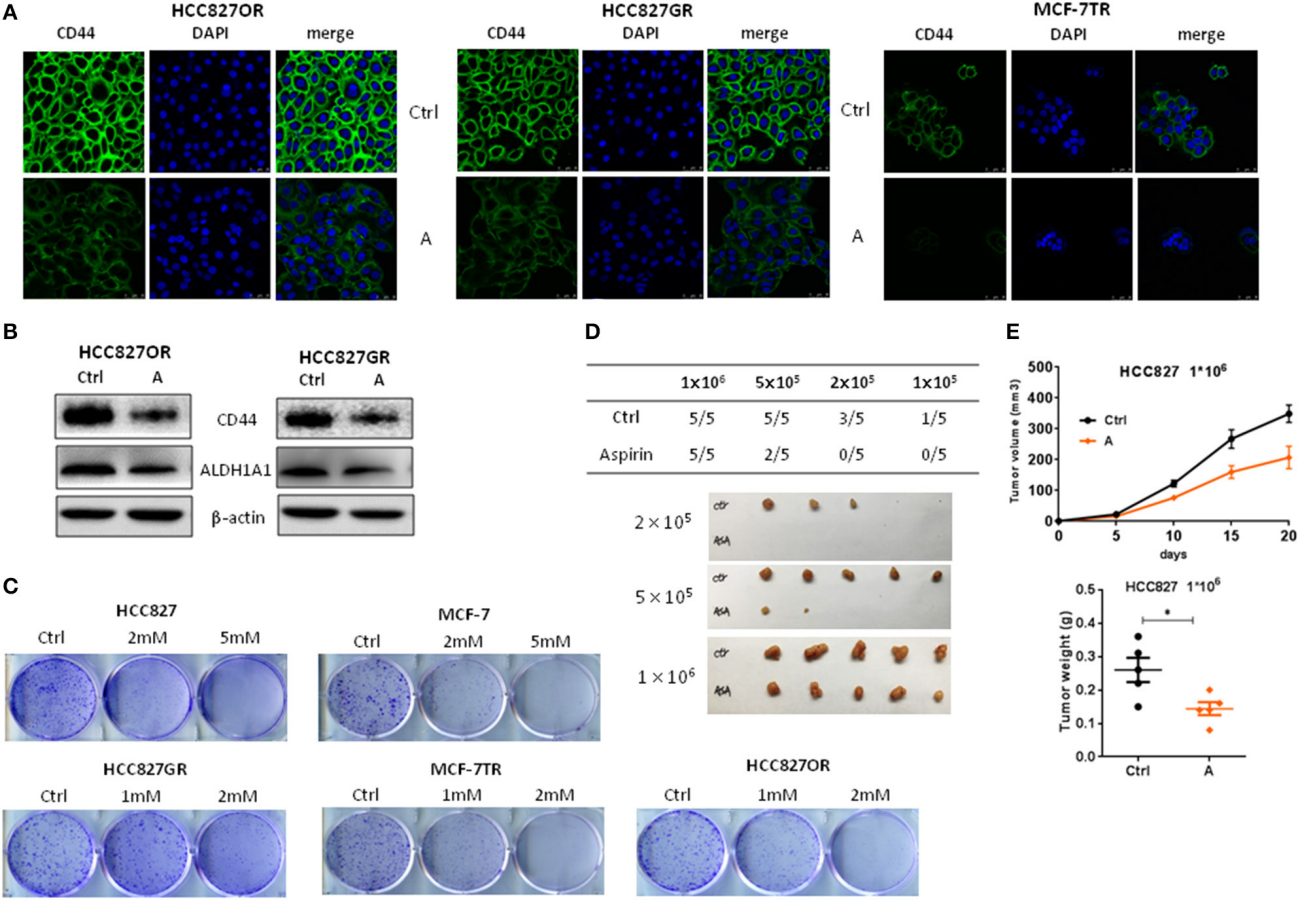
Aspirin suppressed cancer stemness of lung and breast cancers. **(A,B)** The indicated cells were treated with 1 mM aspirin for 72 h. CD44 expression was analyzed by immunofluorescence staining **(A)**. Nuclei were observed with DAPI staining. Scale bar, 50 μm. **(B)** The expressions of CD44 and ALDH1A1 were examined by Western blot. **(C)** Colony formation assay of indicated cells treated with indicated concentrations of aspirin. **(D,E)** Limiting dilution transplantation assay. HCC827 cells were treated with 2 mM aspirin for 12 h prior to transplantation. The indicated numbers of viable HCC827 cells were transplanted subcutaneously into nude mice. The incidence and the photograph of tumors **(D)**. The growth curve and weight of tumors from 1 × 10^6^ HCC827 cells **(E)**. The data are presented as the means ± SEM. Student's *t*-test was used. *P < 0.05.

Cancer stem cells (CSC) are considered to play a pivotal role in therapy resistance and relapse of cancer (23). Next, we sought to examine the effects of aspirin on cancer stemness of lung and breast cancers. Cancer cells with acquired resistance to targeted therapy have been reported with enhanced cancer stemness (24). So, we treated resistant cancer cells with aspirin and then examined the expression of CSC markers CD44 by immunofluorescence analysis. As shown in Figure 4A, the expressions of CD44 in HCC827GR, HCC827OR and MCF-7TR were decreased by aspirin treatment. Western blot also showed that the expressions of CD44 as well as ALDH1A1, which is another well-known cancer stem cell marker, were repressed in HCC827OR and HCC827GR cells treated with aspirin (Figure 4B). Furthermore, colony formation assay showed that aspirin suppressed self-renewal capacity of both sensitive and resistant cells *in vitro* (Figure 4C). To examine *in vivo* tumorigenic capacity, HCC827 cells were treated with aspirin for 12 h, after which 1 × 10^6^, 2 × 10^5^, or 1 × 10^5^ viable cells were implanted subcutaneously into nude mice. Pretreatment with aspirin led to a significant reduction in tumor incidence and tumor volume (Figures 4D,E). Overall, these results demonstrated that aspirin suppressed cancer stemness of lung and breast cancers.

### Aspirin Suppressed NF-κB Signaling Pathway

**Figure 5:**
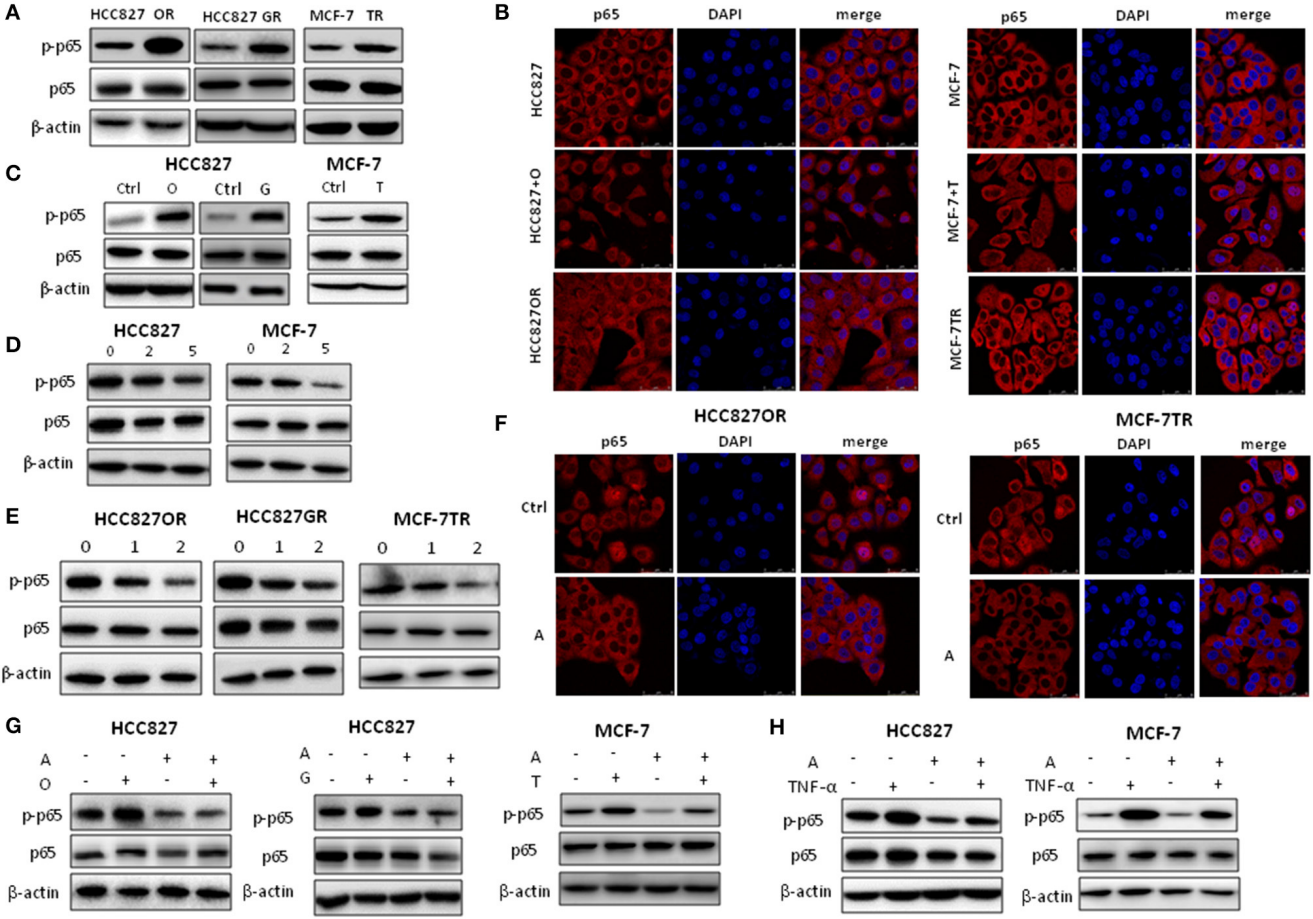
Aspirin suppressed NF-κB activity in cancer cells. **(A)** Western blot analysis of p-NF-κB p65 (p-p65) and NF-κB p65 (p65) in HCC827, HCC827OR (OR), HCC827GR (GR), MCF-7 and MCF-7TR (TR) cells. **(B)** Immunofluorescence staining analysis of p65 in HCC827, HCC827OR, or HCC827 cells treated with 10 nM O for 72 h (left panels) and in MCF-7, MCF-7TR, or MCF-7 cells treated with 2 μM T for 72 h (right panels). **(C)** Western blot analysis of p-p65 and p65 in HCC827 cells treated with 10 nM O or 10 nM G for 72 h (left panels) and in MCF-7 cells treated with 2 μM T for 72 h. **(D)** Western blot analysis of p-p65 and p65 in HCC827 and MCF-7 cells treated with indicated concentration (mM) of aspirin for 72 h. **(E)** Western blot analysis of p-p65 and p65 in HCC827OR, HCC827GR, and MCF-7TR cells treated with indicated concentration (mM) of aspirin for 72 h. **(F)** Immunofluorescence staining analysis of p65 in HCC827OR or MCF-7TR cells treated with 1 mM A for 72 h. Nuclei were observed with DAPI staining. Scale bar, 50 μm. **(G)** Western blot analysis of p-p65 and p65 in HCC827 or MCF-7 cells pretreated with A (2 mM) for 2 h and then subjected to 10 nM O, 10 nM G, or 2 μM T as indicated for 72 h. **(H)** Western blot analysis of p-p65 and p65 in HCC827 or MCF-7 cells pretreated with 2 mM A for 70 h and then subjected to 10 ng/ml TNF-α as indicated for 2 h. β-actin was used as loading control.

Activation of NF-κB signaling has been linked to various aspects of cancer, including inflammation, transformation, proliferation, angiogenesis, metastasis, treatment resistance, and cancer stemness (25, 26). As we previously reported that NF-κB signaling was activated in HCC827GR cells (12), we determined to examine whether aspirin could suppress NF-κB signaling. Consistent with our previous study (12), we found that acquired EGFR TKI-resistant cells HCC827GR and HCC827OR had higher levels of NF-κB activity compared to their parental, sensitive cells HCC827 (Figures 5A,B and Figures S3A,C,E). NF-κB activity was assessed by the levels of phosphorylated NF-κB p65 by Western blot and p65 nuclear translocation by immunofluorescence. Similarly, tamoxifen resistant MCF-7TR cells had a higher level of NF-κB activity compared to parental, sensitive MCF-7 cells (Figures 5A,B and Figure S3E). After HCC827 and MCF-7 cells were treated with targeted drugs gefitinib/osimertinib and tamoxifen for 72 h, respectively, NF-κB activity was also increased (Figures 5B,C and Figures S3A,C,E). These results demonstrated that targeted therapy increased NF-κB activity in cancer cells, which may contribute to the development of acquired resistance. Next, we treated cancer cells with aspirin. As shown in Figures 5D–F and Figures S3B,D,F, aspirin decreased NF-κB activity in both sensitive and resistant cells. These results demonstrated that aspirin suppressed NF-κB signaling. Aspirin also decreased targeted therapy-induced NF-κB activity in HCC827 and MCF-7 cells (Figure 5G). Furthermore, HCC827 and MCF-7 cells were treated with NF-κB activator TNF-α in the presence or absence of aspirin. As shown in Figure 5H, TNF-α increased the level of phosphorylated NF-κB p65 while aspirin abrogated TNF-α-induced p65 phosphorylation, further demonstrating that aspirin suppressed NF-κB signaling.

## Discussion

Acquired resistance to targeted drugs limits the long-term clinical efficacy of these drugs. Therefore, novel therapies such as new drugs or combination of old drugs are needed to overcome or delay the emergence of acquired resistance to targeted drugs. Since new drug development is a time-consuming and expensive process, repositioning old drugs for new indications seems attempting and has set some successful examples (27, 28). In this study, we aimed to reposition FDA-approved drugs to treat lung and breast cancers as well as overcome or delay the emergence of acquired resistance to targeted lung and breast cancer therapies. We searched GEO database for tumor-associated gene signatures (tumor vs. normal) and acquired resistant tumor-associated gene signatures (resistant vs. sensitive) to query the CMap, respectively. Through CMap data mining, we identified 12 candidate drugs. Among these candidate drugs, we found that aspirin has potent antitumor effects on lung and breast cancers and can re-sensitize acquired resistant tumors to targeted therapies as well as delay the emergence of acquired resistance when combined with targeted therapies.

CMap was created to identify compounds that induce a similar or opposite gene-expression signature to diseases of interest. So, it is a useful tool to identify new applications for “old” drug. From GEO, one of the largest gene expression data repositories, we chose lung and breast cancer datasets that contain normal vs. tumor and sensitive/before targeted therapy vs. acquired resistant/after targeted therapy and used selected gene signatures constructed from each dataset to query CMap. Comparing the significant drugs obtained using individual gene signatures, there were 83 overlapping drugs from five normal vs. tumor datasets and 76 from four sensitive vs. resistant datasets. Among these, we found 12 common FDA-approved drug candidates, which have the potential to kill both lung and breast cancers as well as lung and breast cancers with acquired resistance to targeted therapy. Indeed, our results demonstrated that one of these 12 FDA-approved drugs, aspirin, which is widely used and safe, could be repositioned to treat lung and breast cancers.

Epidemiological data have shown that use of aspirin has been associated with lower cancer risk. *In vitro* and *in vivo* studies have also revealed the anticancer activities of aspirin and plausible mechanisms (29–32). Consistently, we found that aspirin inhibited proliferation and promoted apoptosis of lung and breast cancer cells. On the contrary, aspirin has no effects on normal lung and mammary epithelial cell proliferation at concentrations used on lung and breast cancer cells. When combined with targeted drugs gefitinib, osimertinib, or tamoxifen, aspirin had synergistic effects on cancer cell proliferation. More importantly, our results showed that aspirin dramatically increased the sensitivity of resistant tumors to targeted drugs and significantly delayed the emergence of acquired resistance *in vitro* and *in vivo*. In the meanwhile, interestingly, we found that resistant cells were more sensitive to aspirin treatment than parental, sensitive cells in terms of proliferation and apoptosis. Mechanistically, our data showed that resistant cancer cells had higher levels of NF-κB activity compared to their parental, sensitive cancer cells, while NF-κB is reported to be constitutively activated in a wide variety of tumor types including lung and breast cancers compared with their respective normal tissues or cells (33). Aspirin suppressed NF-κB signaling, which could explain the differences in sensitivity to treatment among different cells including normal epithelial cells, sensitive or resistant cancer cells. That is, resistant cancer cells, which have the highest activity of NF-κB, are most sensitive to aspirin, while normal epithelial cells which have the lowest activity NF-κB are least sensitive to aspirin. NF-κB signaling is involved in various aspects of cancer including survival, metastasis, therapy resistance as well as cancer stemness. During the course of targeted therapy, survival tumor cells became resistant, and concomitantly acquired increased cancer stemness and NF-κB activity. Similarly, resistant cells were more sensitive to aspirin treatment than sensitive cells in terms of cancer stemness, which could also be due to increased NF-κB activity in resistant cells. Aspirin was also found to be able to abrogate NF-κB p65 activation induced by targeted drugs or TNF-α. Taken together, we proposed that aspirin could suppress NF-κB p65 signaling to inhibit tumor progression.

In this study, the concentration of aspirin used in *in vitro* experiments was ~2 mM, which is similar to the plasma concentration of salicylate (metabolite of aspirin *in vivo*) observed in rheumatoid arthritis patients. The dose of aspirin used in *in vivo* experiments is 100 mg/kg. According to the Reagan-Shaw method (34), the dose of 100 mg/kg in mice is equal to 8.1 mg/kg in humans, which is the dose that used in clinic to treat inflammation. Thus, the dose in our study is safe for human patients.

## Conclusions

In summary, our data identify aspirin as a potential candidate for combination therapy for lung and breast cancers. There only have been few stage III clinical trials of aspirin plus radiation therapy, chemotherapy, or targeted therapy to treat lung or breast cancer patients. We hope our study provides further molecular rationale and preclinical data to support combination of aspirin with targeted therapy to treat lung and breast cancers.

## Data Availability Statement

All datasets generated for this study are included in the article/Supplementary Material.

## Ethics Statement

All animal experimental procedures were reviewed and approved in accordance with the guidelines for the care and use of laboratory animals at Shanghai Jiao Tong University.

## Author Contributions

LL, MH, TW, and LX designed all the experiments. LL, MH, and TW conducted the experiments, analyzed, and interpreted the results. LX and HC supervised the project. LL and LX wrote the draft manuscript. HC and LX reviewed and edited the manuscript. All authors read and approved the final manuscript.

### Conflict of Interest

The authors declare that the research was conducted in the absence of any commercial or financial relationships that could be construed as a potential conflict of interest.

## Abbreviations

A: aspirin
T: tamoxifen
G: gefitinib
O: osimertinib
CMap: Connectivity map
CSC: cancer stem cell
EGFR TKI: epidermal growth factor receptor tyrosine kinase inhibitor
ER: estrogen receptor
GEO: Gene Expression Omnibus
NSCLC: non-small cell lung cancer
PARP: poly ADP-ribose polymerase.
