# Supplementary material for: Repositioning Aspirin to Treat Lung and Breast Cancers and Overcome Acquired Resistance to Targeted Therapy
Source: Front Oncol. 2020 Jan 14;9:1503. doi: 10.3389/fonc.2019.01503 (PMC6971167; doi:10.3389/fonc.2019.01503)
Supplement: Supplementary file 1 [file Table_1.docx]

**Table S1. List of candidate drugs**

**Table S2. List of antibodies**

| **Table S1. List of candidate drugs** | | | |
| --- | --- | --- | --- |
| fulvestrant | acetylsalicylic acid | glimepiride | thioridazine |
| valproic acid | metformin | sulindac | haloperidol |
| estradiol | sirolimus | troglitazone | triprolidine |

| **Table S2. Antibodies** | | | |
| --- | --- | --- | --- |
| **Target** | **Use** | **Vendor** | **Catalog #** |
| PARP | WB | Cell Signaling Tech | 9542 |
| Cleaved PARP | WB | Cell Signaling Tech | 5625 |
| Caspase-3 | WB | Cell Signaling Tech | 9665 |
| Cleaved Caspase-3 | WB | Cell Signaling Tech | 9664 |
| β-actin | WB | Santa Cruz Biotech | sc-47778 |
| NF-κB p65 | WB, Immunostaining | Cell Signaling Tech | 3195 |
| phospho-NF-κB p65 | WB | Cell Signaling Tech | 5741 |
| CD44 | Immunostaining | Cell Signaling Tech | 9585 |
| ALDH1A1 | WB | Cell Signaling Tech | 54135 |

**Supplementary figure legends**

**Figure S1. The effects of aspirin on the proliferation of normal lung and breast epithelial cells**

Cell viability by CCK8 assay (A, C) and IncuCyte growth curves (B, D) of indicated cells treated with 10 nM osimertinib (O), 10 nM gefitinib (G), 2 mM aspirin (A), 2 μM tamoxifen (T), O+A, G+A, or T+A for 72 hours as indicated. The data are presented as the means ± SEM. Student’s t-test was used. * P < 0.05; ** P < 0.01; *** P < 0.001; **** P < 0.0001.

**Figure S2. The effects of aspirin on the proliferation and apoptosis of normal epithelial, sensitive or resistant cancer cells**

(A, B) Cell viability by CCK8 assay of indicated cell lines treated with indicated concentrations of aspirin for 72 hours. (C) Flow cytometry analysis of apoptosis of indicated cell lines treated with 1 mM aspirin (A) for 72 hours.

**Figure S3. Aspirin suppressed NF-κB activity in cancer cells**

(A) Immunofluorescence staining analysis of p65 in HCC827, HCC827GR or HCC827 cells treated with 10 nM gefitinib (G) for 72 hours. (B) Immunofluorescence staining analysis of p65 in HCC827GR cells treated with 1 mM aspirin (A). Nuclei were observed with DAPI staining. Scale bar, 50 μm. (C-F) Quantification of nuclear p65 in Fig.S3A (C), Fig.S3B (D), Fig.5B (E) and Fig.5F (F). Immunofluorescence quantification of p65 was analyzed by ImageJ. The data are presented as the means ± SEM. Student’s t-test was used. * P < 0.05; ** P < 0.01; *** P < 0.001; **** P < 0.0001.
