# Supplementary material for: Repositioning Aspirin to Treat Lung and Breast Cancers and Overcome Acquired Resistance to Targeted Therapy
Source: Front Oncol. 2020 Jan 14;9:1503. doi: 10.3389/fonc.2019.01503 (PMC6971167; doi:10.3389/fonc.2019.01503)
Supplement: Supplementary file 2 [file Presentation_1.pptx]

## Slide 1
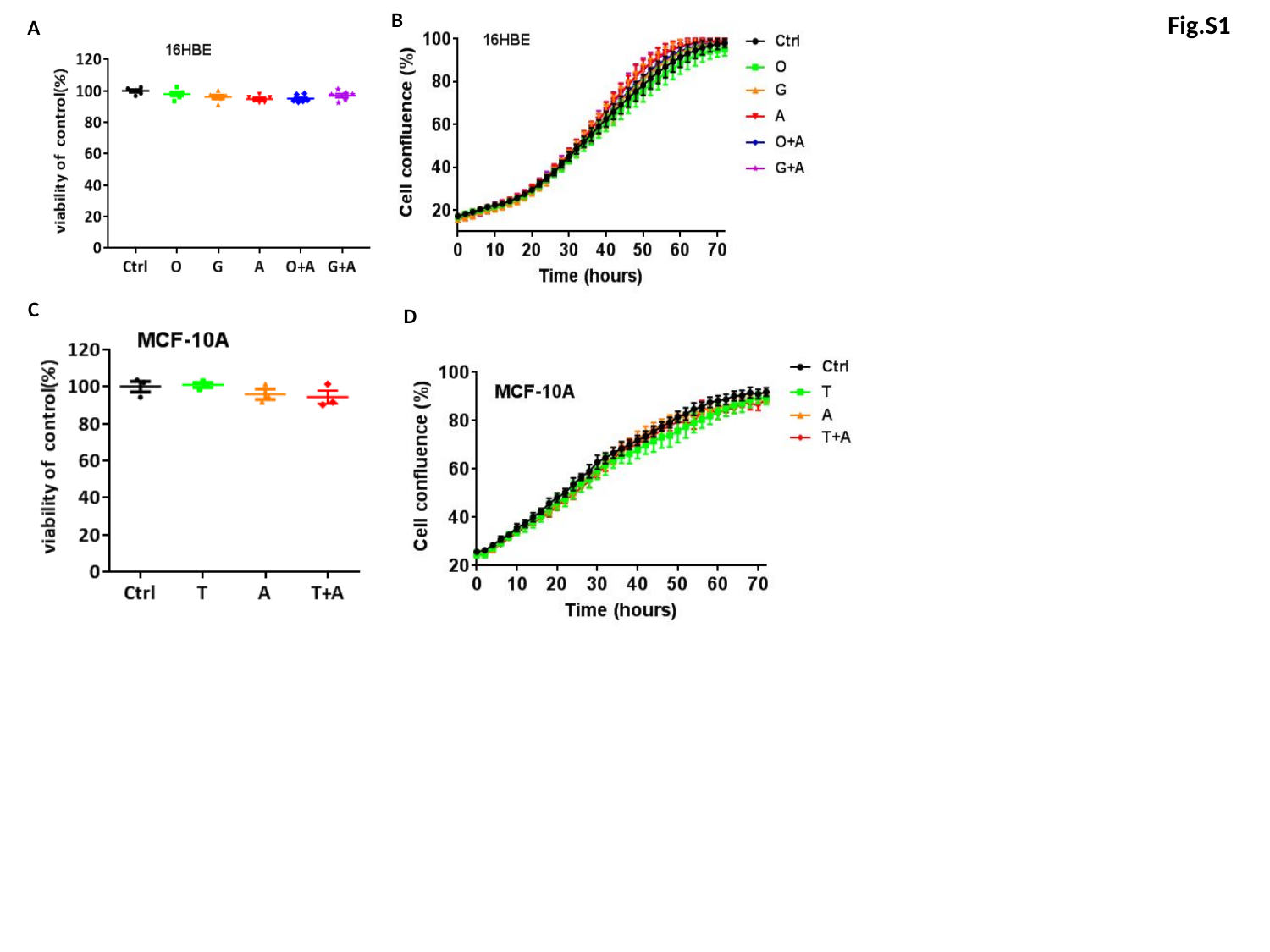

B
Fig.S1
A
C
D

## Slide 2
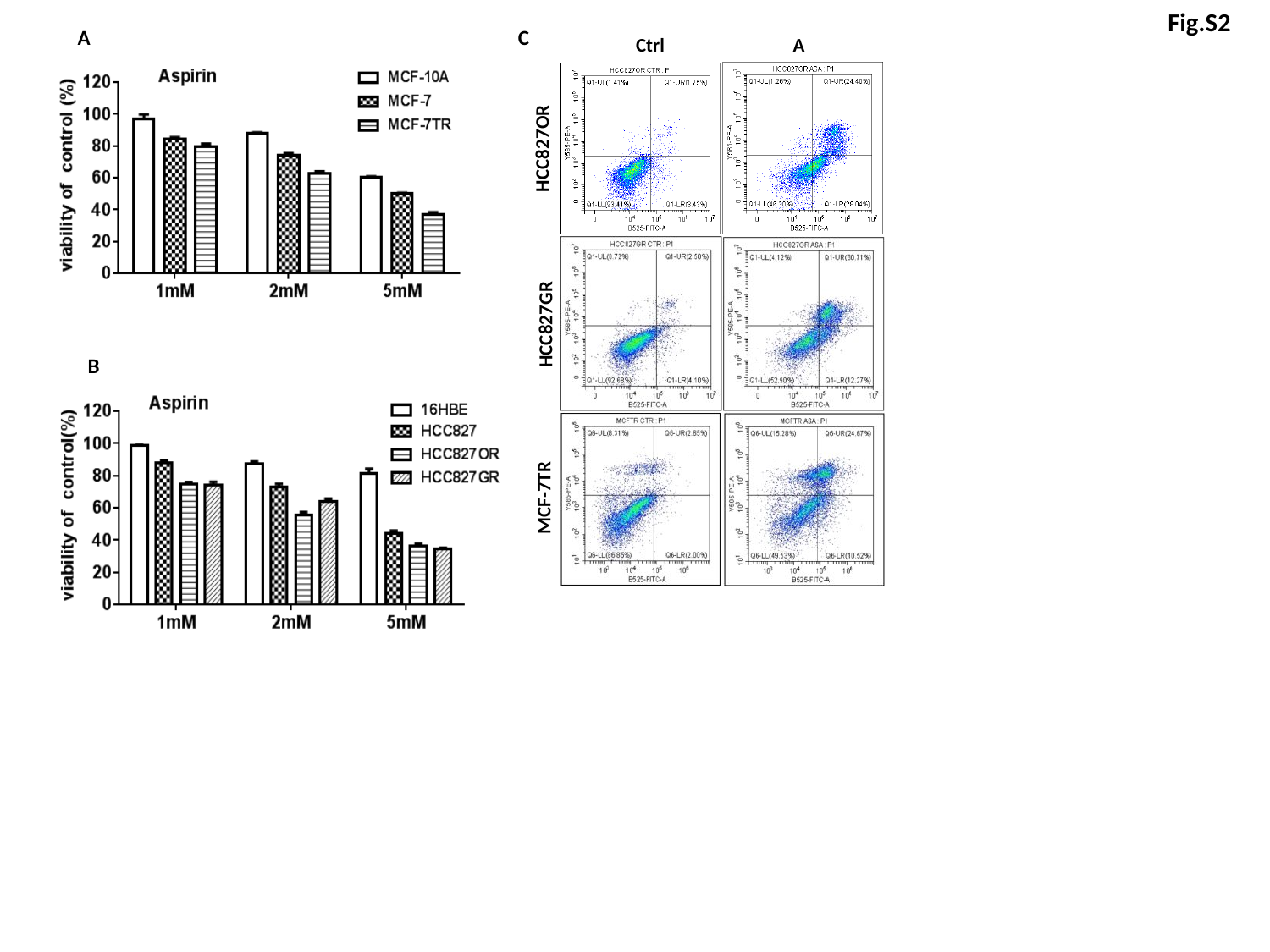

Fig.S2
A
C
Ctrl A
HCC827OR
HCC827GR
MCF-7TR
B

## Slide 3
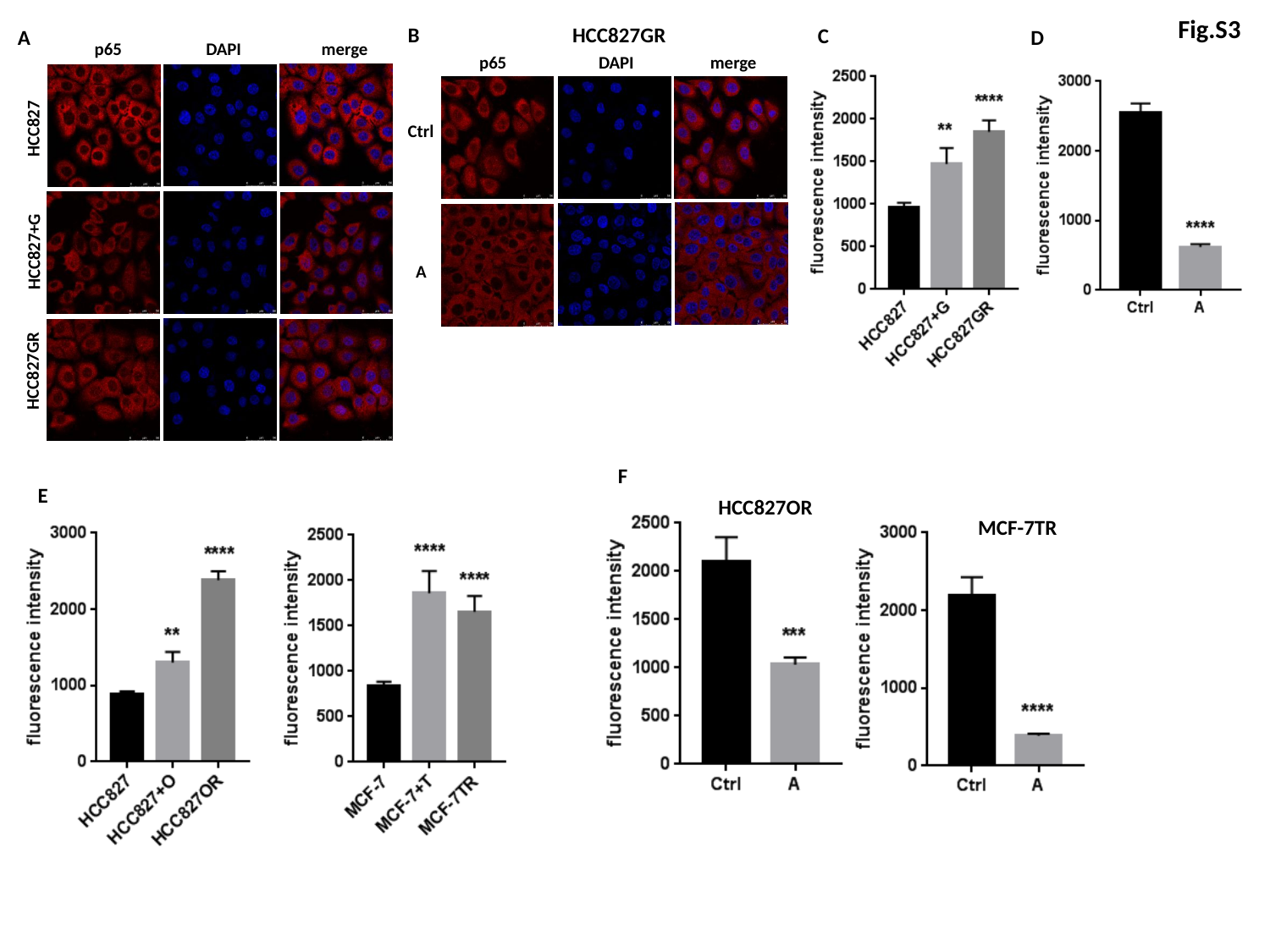

Fig.S3
HCC827GR
p65 DAPI merge
Ctrl
A
B
C
A
D
p65 DAPI merge
HCC827
HCC827+G
HCC827GR
F
E
HCC827OR
MCF-7TR
